# Updates in the Development of ImmunoRNases for the Selective Killing of Tumor Cells

**DOI:** 10.3390/biomedicines6010028

**Published:** 2018-03-05

**Authors:** Sandra Jordaan, Olusiji A. Akinrinmade, Thomas Nachreiner, Christian Cremer, Krupa Naran, Shivan Chetty, Stefan Barth

**Affiliations:** 1 Medical Biotechnology and Immunotherapy Group, Institute of Infectious Disease and Molecular Medicine, Faculty of Health Sciences, University of Cape Town, Cape Town 7700, South Africa; sandrajordaanibms@gmail.com (S.J.); krupa.naran@uct.ac.za (K.N.); 2 South African Research Chair in Cancer Biotechnology, Department of Integrative Biomedical Sciences, Faculty of Health Sciences, University of Cape Town, Cape Town 7700, South Africa; alex.akinrinmadex@gmail.com; 3 Department of Experimental Medicine and Immunotherapy, Institute for Applied Medical Engineering, University Hospital RWTH Aachen, 52056 Aachen, Germany; t.nachreiner@medlife-gmbh.de (T.N.); ccremer@ukaachen.de (C.C.)

**Keywords:** cancer immunotherapy, ranpirnase, human RNases, apoptosis inducers, humanized cytolytic fusion proteins (hCFPs)

## Abstract

Targeted cancer therapy includes, amongst others, antibody-based delivery of toxic payloads to selectively eliminate tumor cells. This payload can be either a synthetic small molecule drug composing an antibody-drug conjugate (ADC) or a cytotoxic protein composing an immunotoxin (IT). Non-human cytotoxic proteins, while potent, have limited clinical efficacy due to their immunogenicity and potential off-target toxicity. Humanization of the cytotoxic payload is essential and requires harnessing of potent apoptosis-inducing human proteins with conditional activity, which rely on targeted delivery to contact their substrate. Ribonucleases are attractive candidates, due to their ability to induce apoptosis by abrogating protein biosynthesis via tRNA degradation. In fact, several RNases of the pancreatic RNase A superfamily have shown potential as anti-cancer agents. Coupling of a human RNase to a humanized antibody or antibody derivative putatively eliminates the immunogenicity of an IT (now known as a human cytolytic fusion protein, hCFP). However, RNases are tightly regulated in vivo by endogenous inhibitors, controlling the ribonucleolytic balance subject to the cell’s metabolic requirements. Endogenous inhibition limits the efficacy with which RNase-based hCFPs induce apoptosis. However, abrogating the natural interaction with the natural inhibitors by mutation has been shown to significantly enhance RNase activity, paving the way toward achieving cytolytic potency comparable to that of bacterial immunotoxins. Here, we review the immunoRNases that have undergone preclinical studies as anti-cancer therapeutic agents.

## 1. Introduction to Targeted Therapy and Immunotoxins

Selective cytotoxicity is the holy grail of targeted anti-cancer therapy. With recent developments in antibody technologies, target-cell selectivity has been demonstrated with monoclonal antibodies and derivatives thereof. Moreover, when these targeting components are armed with apoptosis-inducing effectors, such as chemotherapeutic drugs, small molecule toxins or cytotoxic peptides, the resultant chimeric proteins have exhibited enhanced tumor-specificity in comparison with the toxic agents on their own [1].

The challenge of improving the effective dose regimen of anti-cancer immunotherapy, particularly by reducing immunogenic side effects, has prompted investigation into alternative, humanized substitutes for previously toxin-based effector components [2]. Targeting RNA for degradation by selective delivery of ribonucleases (RNases) presents a mode of cytotoxicity that circumvents some of the challenges faced by standard chemotherapeutic agents. The majority of candidate drugs currently in clinical application disrupt the cell cycle by inducing DNA damage in one way or another, which triggers apoptotic signaling [3]. This can be counterintuitive, as chemo- and radiotherapy are highly mutagenic, which poses the risk of increased resistance in tumors with mutated p53 [4,5]. However, hydrolysis of tRNA by targeted RNases induces cell death via non-mutagenic cell cycle disruption [6].

RNases are comprised of a highly heterogeneous group of enzymes that hydrolyze phosphodiester bonds in mRNA, rRNA or tRNA [7]. They play a role in a wide spectrum of cellular processes and their target(s) may depend on the compartment to which they are deployed, which, in turn, may be influenced by the prevailing metabolic conditions. During conditions of plentiful resources, cells may direct their transcriptional processes to promote protein synthesis and cell proliferation; however, under less abundant conditions, they may lean toward inhibiting biosynthesis and more conservative metabolism [8]. RNases are implicated in the regulation of biosynthesis, by either processing RNA for increased transcription or by degrading RNA to slow resource consumption and even terminate the cell in favor of survival of the organism [9,10,11]. The biological action of select RNases will be discussed later in this review with relevance to their application as immunotherapeutics.

Immunotoxins (ITs), amongst other therapies, have been at the forefront of disease-specific targeted cancer therapy for over three decades. The humanization of ITs represents the fourth and most recent generation of anti-cancer immunotherapeutics, involving the replacement of perennially toxic non-human proteins with enzymes that are non-toxic until delivered intracellularly to the target cell, at which point they abrogate the cell cycle to inhibit tumor growth or, ideally, result in tumor destruction. RNases are widely-studied candidates as inducers of cell death by hydrolysis of cytoplasmic RNA targets. While some RNases inherently exhibit a preference for malignant cells, RNases fused to a cell-targeting component have demonstrated further enhanced tumor specificity [6,12]. This review focuses on the recent developments in the field of recombinant immunotherapeutics with RNases as cytotoxic effector components.

## 2. RNases Studied for Cytotoxicity and Anti-Cancer Activity

### 2.1. Anti-Tumor Activities of RNases

Some RNases, such as members of the RNase A superfamily, exhibit a pyrimidine-specific preference for tumor cell eradication and have been evaluated as potential anti-microbial and anti-cancer agents [11]. Subsequently, bovine pancreatic RNase A [12], bovine seminal RNase [13], Barnase [14], Ranpirnase (RNase I from Rana pipiens) [15], human pancreatic RNase 1 [16], human eosinophil-derived RNase (EDN) [17] and angiogenin (Ang) [18] all exhibit potent toxicity to cancer cells.

### 2.2. Amphibian Ranpirnase: An RNase of Clinical Significance

Ranpirnase is a basic protein consisting of 104 amino acids that was first isolated from the oocytes of Leopard frog Rana pipiens [15,19]. Amino acid sequencing and structural analysis classify this enzyme as belonging to the RNase A superfamily (it shares approximately 30% sequence identity with RNase A) [19]. All three catalytic residues of RNase A are highly homologous in ranpirnase. Structurally, ranpirnase has a more compact conformation owing to disulfide bonds and the presence of a pyroglutamyl residue at the N-terminus, which renders a very stable protein that is resistant to proteolysis [7]. Ranpirnase has the ability to evade inhibition, as demonstrated by the lack of variation in its activity when intracellular levels of RNase inhibitor (RI) are increased or silenced [7]. This evasion of RI is partly attributed to the compact structure of amphibian ribonucleases.

Both the physiological importance and the exact mechanism of its biological activity of ranpirnase are not entirely understood (6); however, its antitumor activity has been demonstrated against several tumor types and has been entirely attributed to its ribonuclease activity [15]. Ranpirnase has been commercially reinvented as Onconase^TM^ (ONC) and has undergone clinical trials as an anti-cancer agent for the treatment of unresectable malignant mesothelioma [20,21].

ONC possesses intrinsic cytotoxicity towards tumor cells independent of fusion to a targeting domain. It has been proposed that internalization occurs through energy dependent endocytosis or through electrostatic interactions between the positively charged ONC protein and electronegative cell membrane of cancerous cells [21]. ONC has an inherent preference for hydrolysis of tRNA, resulting in translational inhibition, and appears to induce apoptosis in a stress-related caspase-9-dependent, p53-independent manner [22]. However, the precise mechanism is still being elucidated. In addition, several other RNAs are targeted by ONC that are involved in cell cycle control, gene regulation by non-coding RNAs and transcription factors, as revealed by global transcriptional analyses using expression arrays [23,24,25].

Phase I and II clinical trials evaluating ONC, as for the treatment of non-resectable malignant mesothelioma, reported dose-limiting toxicity and adverse side-effects in some patients [26]. However, positive responses in a number of patients—as well as the fact that ONC has been reported to act synergistically with cepharanthine, cisplatin, doxorubicin and rosiglitazone [7] to achieve enhanced tumor eradication by multi-compound therapy—promoted ONC to potential new drug status pending phase IIIb clinical trials. The phase IIIb trial in patients with unresectable malignant mesothelioma was concluded in 2007 and were not particularly successful based on number of patient deaths (316 or 428 enrolled), with additional reports of some adverse side-effects such as peripheral edema, renal insufficiency and allergic reactions in some patients [27], which may be the result of cytotoxicity of ONC in non-tumor cells. More recently, pre-clinical testing of ranpirnase recombinantly fused with an antibody targeting EGFR-positive tumors demonstrated no dose-limiting toxicity and an absence of off-target effects [28], suggesting that recombinant targeted versions of this RNase may provide a more promising approach.

### 2.3. Targeted RNases as Anti-Cancer Agents

The conditional cytotoxicity of RNases has been exploited (and enhanced) for selective depletion of cancer cells by conjugation or fusion of the RNase to a disease-targeting antibody, generating a chimeric antibody-RNase referred to as an immunoRNase. The antibody or targeting domain of this immunoRNase is selected, based on two features: (a) the relative expression of its target biomarker or receptor on the surface of diseased cells in relation to healthy tissues (minimizing collateral toxicity), and (b) the ability of the cell-surface target to induce receptor-mediated endocytosis of the immunoRNase (achieving payload internalization, as RNA is an intracellular target).

RNases as cytotoxic effectors in disease-specific immunoconjugates/immunofusions have previously been extensively reviewed [10,11,13,29,30,31,32,33,34] and several immunoRNases (Table 1 and Table 2) have been shown to exhibit anti-cancer activities, shown as 50% growth inhibitory coefficients (IC_50_).

Fusion of ONC to a disease-specific antibody can direct its cytotoxicity to tumors with greater potency than the RNase in isolation. Newton et al. [50], as well as Weber et al. [51], have produced fusion proteins comprising of an anti-CD22 and ONC targeting B cell lymphomas. These showed significant in vitro and in vivo efficacy with as much as 104-fold increased toxicity in non-Hodgkin’s lymphoma cells in comparison to wild-type ranpirnase, with minimal side effects.

### 2.4. Humanized Immunofusions: More of the Good, Less of the Bad

The immunogenicity of mouse-derived antibodies in clinical applications is one of the reasons humanized ITs, most recently known as targeted human cytolytic fusion proteins (hCFPs), are under investigation as an alternative to fusions of non-human domains [52]. As described earlier in this review, effectors based on bacterial or plant protein toxins also frequently induce immunogenicity, as well as being accompanied by toxic off-target effects due to their native infectious properties. Combining humanized antibody derivatives with human apoptosis-inducing enzymes as therapeutic fusions is anticipated to be less immunogenic in human patients. Furthermore, targeted human cytolytic proteins lack inherent cell binding and membrane translocation capacity: hence, they are dependent on their antibody-moiety for delivery and cytolytic action.

Recently, hCFPs comprising microtubule-associated protein tau (MAP tau) in fusion with single chain antibody fragments (scFv) targeting lymphoma [53], rhabdomyosarcoma (RMS) [54,55] and triple negative breast cancer [56] were investigated. The high dose tolerance in mice was suggestive of low off-target toxicity, while potency of these hCFPs was comparable to that of Pseudomonas exotoxin A-based ITs. Granzyme B (GrB), a cytolytic protein unleashed upon cells that are designated for elimination by cytotoxic T-lymphocytes [57], has also recently featured as a hCFP effector. The H22(scFv)-GrB hCFP targeting the CD64 receptor, a marker of a monocytic subtype of acute myeloid leukemia (AML), exhibits selective and specific cytotoxicity against CD64+ AML cells, while sparing CD64-cells [58]. The disease specificity demonstrated with these hCFPs in animal studies, together with the expected low immunogenicity of their humanized components, indicate potential suitability of hCFPs for clinical application in human patients.

### 2.5. Human RNases with Therapeutic Potential

Several RNases have been shown to exhibit conditional anti-tumor activity [7]; however, the ability of amphibian and mammalian RNases to penetrate cells can produce off-target toxicity and, as described earlier, induce an immunogenic response to their foreign epitopes in human patients. Human RNases on their own possess limited cytotoxicity but have been shown to exhibit enhanced and more specific cytotoxicity when fused with a disease-specific antibody. The increasing interest in immunofusions of human RNases to enhance their cytotoxicity, largely spear-headed by researchers such as Susanna Rybak, Guiseppe D’Alessio, Maria Vilanova and Thomas Schirrmann, spearheaded immunoRNases to the forefront of developing hCFP technology.

#### 2.5.1. Human Pancreatic RNase or RNase 1

Contrary to its name, human pancreatic RNase (HP-RNase) is not exclusively produced in the pancreas and its expression is widely distributed throughout a range of tissues and organs [59], thus alluding to its roles in processes other than digestion. The mature enzyme, consisting of 128 amino acids [60], appears to possess no inherent toxicity, and its physiological activity resembles that of bovine pancreatic RNase [61], more than other human RNases such as eosinophil derived neurotoxin (EDN) and angiogenin [40]. HP-RNase 1 efficiently hydrolyses double-stranded RNA (dsRNA) at the 3′-end of pyrimidine bases, with a substrate preference for poly(C) over poly(U), but can also cleave poly(A) [62,63]. It is also hydrolyzes the RNA component of hybridized DNA:RNA with much higher activity than RNase A [64]. HP-RNase has been shown to exhibit specific tumor suppression in fusion with a range of disease-specific ligands and antibodies (Table 2). De Lorenzo et al. [2] generated a fusion of HP-RNase1 and hErbB2, demonstrating selective cytotoxicity towards carcinomas expressing the ErbB2 receptor. Tumor-targeting immunofusions bearing HP-RNase1 have been shown to specifically bind tumor cells, undergo internalization and induce apoptosis, in numerous studies [40,44,46].

#### 2.5.2. Eosinophil-Derived Neurotoxin or RNase 2

Components of eosinophil granules were initially recognized by M. H. Gordon to induce neuronal degeneration and a 18.4 kD isolated protein was thus later designated eosinophil derived neurotoxin (EDN) [65]. EDN, or human RNase 2, possesses high sequence identity with bovine RNase A [66,67] and exhibits comparable RNase activity [68]. It has been postulated to possess anti-viral properties as a result of its RNase activity [69], but no inherent cytotoxicity for healthy mammalian somatic cells [67].

EDN has been included in single-chain immunofusions targeting the human transferrin receptor [70], and these studies demonstrated selective in vitro cytotoxicity in erythroid leukemia, squamous cell carcinoma, melanoma, breast carcinoma and renal cell carcinoma cell lines [17,40,41]. Cytotoxicity was found to be highly target-specific, whereas EDN free of a targeting domain did not exhibit comparable toxicity, a feature crucial in targeted therapy.

#### 2.5.3. Angiogenin or RNase 5

The 14.4 kD human ribonuclease angiogenin (Ang), also known as human RNase 5, is another member of the RNases A superfamily. It was first described in 1985 by Vallee et al. [71] as a secreted protein present in conditioned media of cultured adenocarcinoma cells [72]. Ang shares approximately 33% sequence homology with other members of the RNase superfamily [73] and 65% identity with bovine RNase A [74]. As its designation indicates, Ang has been shown to contribute to angiogenesis, particularly in tumors. While this might seem somewhat contradictory with the evidence of its anti-cancer efficacy, the answer lies in its conditional, compartment-specific activity.

**Figure 1:**
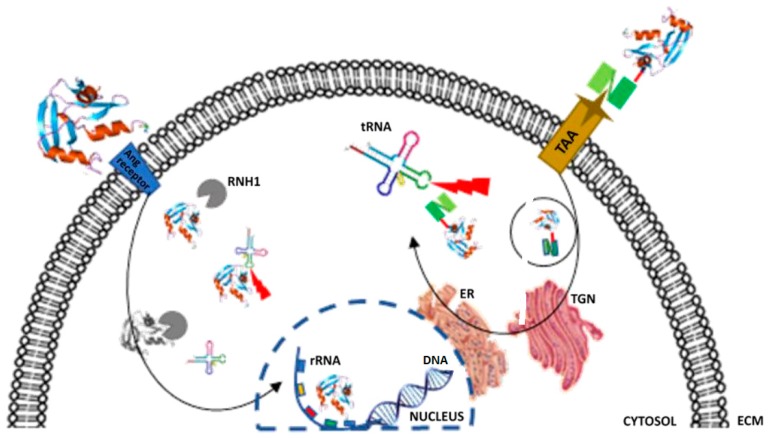
Mechanism of action following targeted Angiogenin delivery to a cell. Angiogenin binds to a membrane receptor (blue wedge), localizes to the nucleus and engages in rRNA processing. Under stress conditions, Ang is found more prolifically in the cytosol, where it degrades tRNA and blocks biosynthesis. An Ang-based hCFP is designed to be selectively internalized via a disease-associated antigen and delivered to the cytosol, where it degrades tRNA similarly to free cytosolic Ang. Both free Ang and immuno-Ang are sensitive to cytosolic RNH1 inhibition.

Typically, secreted extracellular Ang enters the cell via a 170 kD cell-surface receptor, upon which a nuclear localization signal (RRRGL) at positions 31–35 facilitates nuclear translocation. In the nucleus, Ang acts as a transcription factor for biosynthesis and processes rRNA, earning its designation as a potent inducer of angiogenesis (Figure 1) [75,76,77]. Putatively, due to the high metabolic needs of cancerous cells, Ang is highly upregulated in tumors, where it contributes to neovascularization and proliferation [78].

Conversely, under conditions of cellular stress such as low resource availability (starvation), Ang is expelled from the nucleus into the cytosol. Here, Ang hydrolyses tRNA at the exposed anti-codon loop, producing cleaved fragments referred to as tRNA-derived stress induced RNAs (tiRNA) [8,74,79]. These tiRNA fragments form complexes, which ultimately modify gene expression and inhibit translational initiation, thus abrogating protein synthesis and promoting induction of apoptosis [8,72].

Stress-induced tRNA cleavage has not only been described in mammalian cells, but it also has been described in various bacteria and fungi [80,81], indicating an evolutionary conserved mechanism of resource preservation and protein recycling during stress, promoting organism survival under nutrient-poor conditions, potentially at the expense of individual energy-demanding cells [82].

The stress-related accumulation of Ang in the cytosol would be mimicked by internalization and cytosolic delivery of an Ang-based immunoRNase. While sub-cellular routing of immunoRNases still requires further optimization, Ang-based hCFPs have been shown to effectively deplete solid and hematological tumors in association with a range of antibodies and ligands [41,42,45,47,48]. Krauss et al. [36] have successfully generated a fusion of an anti-CD22 scFv to human Ang, which selectively targets and depletes malignant C22-positive B-cell lymphoma with an IC_50_ of 56 nmol/L. This construct was easily purified to homogeneity and seemed to retain full ribonucleolytic activity. Most recently, Gresch et al. [83] have demonstrated the significant increase in the pro-apoptotic efficacy of an anti-CD89 (scFv)- AngGGRR in AML-derived target cells and leukemic blasts, compared to the conventional IT, ETA. Table 2 comprises more examples of RNase-based fusions and immunoRNases.

As previously mentioned, sub-cellular routing presents one of the greater challenges influencing hCFP cytotoxicity, predominantly when the catalytic target is cytoplasmic or nuclear. In the case of enzymatic effectors, which form the basis of a large proportion of hCFPs including immunoRNases, another downstream challenge is the susceptibility of the hCFP to its enzyme effector’s cellular inhibitor(s). Delivery of the immunoRNase to its target compartment may be neutralized by the presence of endogenous RNase inhibitors that bind to and block the enzyme. Overcoming cellular inhibition has been the aim of more recent hCFP engineering developments.

However, Schirrmann et al. [84] were not able to demonstrate that IgG-fusions of HP-RNase 1 had any significant tumor suppressive effect, even though RNase inhibitor did not appear to be the obstructing factor. Despite the introduction of putative endosomal cleavable linkers, it remains unclear whether the HP-RNase 1 component of IgG-RNases translocated across the cytosolic lipid membrane bilayer and reached the cytosol in its active form. Furthermore, the group failed to explore different constructs that may play an important role in RNase activity and affect kinetics, as shown by Menzel et al. [46]. Moreover, it could be that the IgG platform is not suitable in certain contexts, which could possibly fail in the intracellular processing pathway after internalization.

The work of Schirrmann et al. highlights the complexity of cell biology, and the exact mechanisms of immunoRNase cytotoxicity are yet to be completely elucidated.

### 2.6. RNase Inhibitors Limit ImmunoRNase Potency

Pancreatic-type RNase A, RNases 1, 2 and 5 (angiogenin) are regulated by intracellular ribonuclease inhibitor (RI), encoded by the *RNH1* gene [85]. While the biological role of RI remains unclear, its cytosolic prevalence may imply a compartmentalized regulatory function with respect to RNase activity [86]. Cytosolic RNA hydrolysis is invariably detrimental to protein biosynthesis and cell growth, whereas nuclear RNases tend to contribute to RNA processing and replication. It has been proposed that it is not the localized levels of the RNase, but rather the compartment-specific levels of RI that regulate ribonuclease activity within the cell.

The cytotoxicity of immunoRNases is dependent on their cytosolic ribonucleolytic activity and ribosome inactivation [87]. The presence of cytosolic RI thus presents a major obstacle for apoptosis induction in target cells by RNase-based hCFPs. The amphibian ONC, described earlier in this review, has a low binding affinity for RI, whereas RNase A has a relatively high affinity [86,88]. Bovine seminal RNase, the only naturally dimerizing member of the RNase A superfamily, is more inhibition-resistant in its dimerized state than as a monomer [89]. The next progression in hCFP drug development has thus been the generation of RI-resistant RNases. Once the region of interaction between RNase and RI has been identified (by structural analysis of the RNase-RI complex), steric obstruction of the binding region may be conceptualized either by introducing large, charged residues or introducing Cys residues that trigger dimerization and has been shown to reduce RNase-RI affinity [87].

## 3. Angiogenin Mutants and Inhibition by RI/RNH1

### RNase Mutants/Variant Designed to Be Resistant to RI

Both ribonuclease inhibitors and members of the RNase A superfamily exhibit high cross-species homology, while the regions involved in inhibitor binding appear to share similarities [85].

The X-ray crystal and NMR solution structures of the RNase-RI complex can be used to earmark amino acids in the interacting region, which may contribute to the stability of the complex. This information can guide the introduction of point mutations, which may, in turn, destabilize the RNase-RI complex. The RNase A variant G88R, featuring a substitution of Gly88 which resides in the RI binding domain, is more cytotoxic than the wild type RNase A [90]. The introduction of an arginine residue at this position appears to act as an obstructing factor of RNase affinity for RI and thus reduces sensitivity of RNase to inhibition. An RI-resistant variant of HP-RNase developed by Quintessence Bioscience Inc (designated QBI-139) went into Phase I clinical trials in 2010 [91].

Human Ang, compared with other members of the RNase A superfamily, possesses weak ribonuclease activity with a substrate affinity 104–106 fold lower than that of RNase A [92]. A large, obstructive glutamine residue residing within the active site of the enzyme (Q117) is ascribed for the weak interaction with its RNA substrate. Furthermore, non-nuclear Ang activity is limited by the cytosolic prevalence of endogenous RI, which binds and inactivates Ang with high affinity [93,94]. The cytotoxicity of Ang-based fusion proteins is thus hypothesized to be less reliant on delivery of large quantities of Ang to the cytosol, but rather delivers inhibition-resistant Ang with a higher substrate affinity [87].

High performance computing has facilitated high-resolution simulations of dynamic protein interactions to more precisely model RNase inhibition [95]. These simulations provide in-depth information concerning amino acids crucial to the RNase-RI complex, thus identifying specific candidates for modification of this interaction. Recently, Cremer et al. [47,48,96] have designed variants of Ang fused with CD64-specific scFv (H22), which have 30-fold higher ribonuclease activity relative to the wild type Ang, due to amino acid substitutions, which reduce their sensitivity to RI and enhance their substrate affinity in vitro and in vivo [97].

## 4. Conclusions

Maximizing apoptotic potency while minimizing collateral toxicity is a qualitative function of the substrate and the choice of effector targeting said substrate. The effector’s cytotoxicity, in turn, is dependent on its own catalytic activity, as well as its resistance to inhibition. RNases play a crucial role in cellular biosynthesis and are tightly regulated by endogenous inhibitors, particularly in subcellular compartments where their unchecked activity may be detrimental to cell survival. ImmunoRNases are not impervious to inhibitors and thus are only sustainably effective if they exhibit increased resistance to inhibition [98].

Angiogenin has been applied as cytotoxic effector in immunofusions for over 30 years and, more recently, variants of the enzyme with reduced affinity for RNase inhibitor have been shown to exhibit enhanced cytotoxicity. The human RNase is a potent antiproliferative agent when coupled to a tumor cell-specific binding domain. This and other novel modified immunoRNases with improved properties pave the way towards an entirely human immunotherapeutic agent with comparable, and eventually even superior, toxicity to that of traditional immunotoxins.

While several therapeutic monoclonal antibodies are in various stages of clinical trial or application for targeted cancer therapy, immunoRNases are still restricted to testing at a pre-clinical level. RNases as candidate cytotoxic effector domains in hCFPs are promising in terms of their potency and lack of immunogenicity; however, their application is also subject to several challenges. Computational simulation of enzyme–substrate interactions and information-guided enhancement of cytotoxic enzyme activities may address some of these challenges currently experienced with respect to in vivo efficacy of hCFPs.

Although the efficacy of these agents is being progressively optimized by increasing enzyme activities, reducing sensitivity to inhibition and improving intracellular delivery by means of architectural modifications (such multivalency and the addition of cleavable linkers), clinical exploitation of immunoRNase technology is still presently limited by production scaling as well as production costs [99]. However, the use of appropriate mammalian production systems appears to be overcoming production constraints and may soon yield clinically viable immunoRNase therapeutic agents.

## Figures and Tables

**Table 1:** Non-humanized immunoRNases.

ImmunoRNase ᵠ	Target Antigen	Effector RNase	Mode of Conjugation	Cancers Tested *	IC_50_ (nM)	Ref.
454A12-RNase	Transferrin receptor	RNase A	SPDP coupling	GB, Leuk	<260	[12]
5E-9-RNase	Transferrin receptor	RNase A	SPDP coupling	GB, Leuk	<260	[12]
T101-RNase	T-cell antigen CD5	RNase A	SPDP coupling	GB, Leuk	~120	[12]
EGF-RNase	EGF receptor	RNase A	SPDP coupling	SCC, BC, SCLC	300–1000	[35]
*Rap*LRI-SGIII(scFv)	CD22	*R.pipiens* RNaseI	Rec. fusion	Burkitt’s Lym	132–185	[36]
*Rap*LRI-SGIII (diabody)	CD22	*R.pipiens* RNaseI	Rec. fusion	Burkitt’s Lym	3–20	[36]
4D5(scFv)-dibarnase	HER2	Barnase	Rec. fusion	BC	2.4–4.1	[37]
scFvA33T1	GPA33	RNase T1	Rec. fusion	CC, PC	300	[38]
Ranpirnase- αEGFR(scFv)	EGF receptor	Ranpirnase	Rec. fusion	SCC	120–>360	[28]

ᵠ Recombinant fusion proteins, such as immunoRNases, expressed in an *E. coli* expression system are purified from inclusion bodies and such bacterial expression systems are known for their high protein yields [39]. * SCC, squamous cell carcinoma; BC, breast cancer; SCLC, small cell lung carcinoma; Mel, melanoma; GB, glioblastoma; Leuk, leukemia; Burkitt’s Lym, Burkitt’s Lymphoma; RCC, renal cell carcinoma; CC, colorectal carcinoma; PC, pancreatic carcinoma.

**Table 2:** Humanized ImmunoRNases as human cytolytic fusion proteins (hCFPs) in preclinical development.

ImmunoRNase	Target	Effector RNase	Mode of Conjugation ^Δ^	Cancers Tested *	IC_50_ (nM)	Ref
EDN-sFv	Transferrin receptor	EDN	Rec. fusion	Leuk.	0.2–1.0	[17]
EDN- CD71	Transferrin receptor	EDN	Rec. fusion	Mel, RCC, BC	1.2–8	[40]
RNase1-CD71	Transferrin receptor	HP-RNase1	Rec. fusion	Mel, RCC, BC	5–10	[40]
Ang-E6	Transferrin receptor	Angiogenin	Rec. fusion	Glioma, TNBC	15, 45	[41]
EGF-Ang	EGF receptor	Angiogenin	Rec. fusion	SCC	12.5–45	[42]
CL-RFN89	FGF receptor	HP-RNase1	Insert. fusion	Mel	60–460	[43]
hpRNase1-hIL-2	IL-2 receptor	HP-RNase1	Rec. fusion	act. T lymphocytes	20	[44]
hERB2-hRNase	ErbB-2 receptor	HP-RNase1	Rec. fusion	BC	12.5–60	[2]
MJ7(scFv)-Ang	CD22	Angiogenin	Rec. fusion	Burkitt’s Lym	<1000	[45]
MLT7(dsFv)-Ang	CD22	Angiogenin	Rec. fusion	Burkitt’s Lym­	~100	[45]
αCD30(scFv-Fc)-RNase	CD30	HP-RNase1	Rec. fusion	Lymphoma	3.3	[46]
H22(scFv)-Ang	CD64	Angiogenin	Rec. fusion	Leuk, M1 macrophages	10 ± 2.7	[47,48]
4LB5-HP-RNase	NCL	HP-RNase	Rec. fusion	TNBC	20–70	[49]

^Δ^ Rec.fusion, recombinant fusion; Insert. fusion, insertional fusion. * SCC, squamous cell carcinoma; BC, breast cancer; SCLC, small cell lung carcinoma; Mel, melanoma; GB, glioblastoma; Leuk., leukemia; Burkitt’s Lym, Burkitt’s Lymphoma; RCC, renal cell carcinoma; CC, colorectal carcinoma; PC, pancreatic carcinoma, TNBC, triple negative breast cancer.
